# Neurogenic Appendicitis: A Reappraisal of the Clinicopathological Features and Pathogenesis

**DOI:** 10.3390/diagnostics12061386

**Published:** 2022-06-03

**Authors:** Mahmoud Rezk Abdelwahed Hussein, Ali Al Bshabshe, Ahmed Abdelsatar Elhakeem, Mahmoud Kamal Elsamman

**Affiliations:** 1 Department of Pathology, Assiut University Hospitals, Assiut 71515, Egypt; 2 Department of Medicine, College of Medicine, King Khalid University, Abha 62527, Saudi Arabia; albshabshe@yahoo.com; 3 Department of Pathology, Alazhar University, Cairo 11884, Egypt; ahmad.elhakeem2011@yahoo.com; 4 Department of Internal Medicine, Faculty of Medicine, Sohag University, Sohag 82725, Egypt; elsamman73@gmail.com

**Keywords:** appendix, neurogenic, neuroinflammation

## Abstract

In 1921; Masson and Maresch first coined the term “neurogenic appendicitis (NA)” to describe “neuroma-like” lesions in the appendix. To date, our knowledge about NA is limited; therefore, we conducted a comprehensive analysis of the literature (1921 to 2020) to examine the clinicopathological features of NA. We also addressed the pathophysiology of acute abdominal pain and fibrosis in this entity. We performed a meta-analysis study by searching the PubMed database, using several keywords, such as: “appendix,” “neurogenic,” “obliterative,” “neuroma,” “fibrous obliteration,” “appendicopathy,” and “appendicitis.” Our study revealed that patients with NA usually present clinically with features of acute appendicitis, bud2t they have grossly unremarkable appendices. Histologically, the central appendiceal neuroma was the most common histological variant of NA, followed by the submucosal and intramucosal variants. To conclude, NA represents a form of neuroinflammation. The possibility of NA should be considered in patients with clinical features of acute appendicitis who intraoperatively show a grossly unremarkable appendix. Neuroinflammation and neuropeptides play roles in the development of pain and fibrosis in NA.

## 1. Background

The vermiform (worm-like) appendix is a tubular extension of the cecum. It originates from a primordial structure known as the cecal diverticulum. It becomes visible during the eighth week of gestation. Although the exact functions of the appendix are not clear, it seems to be involved in the mucosal immune response. Histologically, the appendix is composed of four layers, including the mucosa, submucosa, muscularis propria, and serosa. The wall of the appendix contain endocrine and neuroglial cells, as well as nerve fibers [1,2].

The term “neurogenic appendicopathy(NA)” has been coined for patients with clinical symptoms reminiscent of acute appendicitis, but whose appendectomy specimens lack the histological features of acute inflammation. To date, little knowledge is available about NA [3,4,5].

To gain more insights into this enigmatic entity, we conducted a literature search and analysis of the relevant investigations about NA. Our current study addressed several issues about the following aspects of NA, including: (i) the historical and clinical features, (ii) the enteric nervous system, (iii) the pathogenesis, (iv)the roles of NPs and neuroplasticity, (v) the pathological features and the histological variant, (vi) the evolving and regressing phases, (vii) neuroinflammation, and (viii) the underlying mechanisms of acute abdominal pain and fibrosis in NA.

### 1.1. The Historical and Clinical Aspects of NA

In 1921, Masson and Maresch first applied the term “NA” to describe “neuroma-like” lesions in appendectomy specimens [6]. As the lesions were lacking the features of acute inflammation (non-acute appendicitis), Hofler introduced the term “neurogenic appendicopathy” [7]. NA is also known as fibrous obliteration of the appendix, neuroimmune appendicitis, non-acute appendicitis, chronic obliterative appendicitis, or appendiceal neuroma. The term “chronic appendicitis” is one of several terminologies used to describe cases of NA with fibrosis, but lacking an acute inflammatory reaction. NA represents from 0.16% [8] to17.1% of all cases of surgically treated appendicitis [5,9,10]. It is more common in females than in males. It is common in the third decade (27.8 ± 12 years) of life [8], and after 45 years of age, but uncommon in the first decade of life [7]. 

To date, the concept of NA is not uniformly accepted in medicine, with only a fraction of surgeons and pathologists recognizing it as a valid diagnosis. This is due to the lack of consensus relating to the diagnostic criteria of NA [11,12].There are no specific radiological signs that define this condition [5,9]. Clinically, it is hard to distinguish between NA and acute appendicitis [7,13]. A definitive diagnosis can only be made by histological examination of the grossly inconspicuous appendectomy specimens. Franke et al. examined 282 appendectomy specimens. They found NA in 3.8% of patients with acute appendicitis and 47% of those with a negative appendectomy. The authors indicated that neither clinical history nor physical examination can preoperatively separate NA from acute appendicitis [14,15]. The clinical features of NA varied from nonspecific symptoms (anorexia, nausea, and vomiting) to features frequently seen in acute appendicitis, such as abdominal pain, guarding, rigidity, and rebound tenderness in the right iliac fossa [5,14,15]. These symptoms reflect the presence of abundant numbers of neurofibers, together with the increased release of several neuropeptides (NPs) [7,13]. The endocrine cells within the hypertrophied nerve bundles in NA may be the site of origin of the appendiceal neuroendocrine tumors [16].

### 1.2. The Enteric Nervous System

The enteric nervous system originates from the neural crest. It consists of thousands of small ganglia and their interconnecting nerve fibers that populate the gut wall. The ganglia are composed of neurons and glial cells, and they are similar in structure to those in the central nervous system. The bundles of nerve fibers consist of the axons of enteric and extrinsic neurons. The ganglia include two sets: the submucosal ganglia and the myenteric ganglia, which are located amid the external muscle layers. There are complex interactions between the enteric nervous system and both the intrinsic immune and endocrine systems [17]. Structurally and neurochemically, the enteric nervous system represents a brain unto itself [18,19,20,21,22,23].

In the appendix, the crypt epithelium contains flask-shaped or spindle-shaped endocrine cells, arranged singly or as aggregates among the epithelial cells [24]. These cells are reactive for the neuroendocrine markers, such as chromogranin and synaptophysin. They are also reactive for several amines and polypeptide substances, such as substance P (SP), serotonin, somatostatin, and enteroglucagon [25]. Ultrastructurally, these cells have cytoplasmic neurosecretory granules (NSGs) [26]. The lamina propria, just beneath the mucosal crypts of the appendix, contains numerous mucosal neuroendocrine complexes (neuroendocrine ganglia). The latter is composed of a well-developed mucosal nerve plexus which represents an admixture of ganglion cells, Schwann cells, axons, and neuropil. They uniquely entangle endocrine (neurosecretory) cells [27]. The neuroendocrine ganglia are interconnected by S100, SP, and neuron-specific enolase (NSE) positive neural fibers. These cells communicate with the enteric nervous system. The neuroendocrine ganglia modulate neural communications through serotonin mediators. 

### 1.3. The Pathogenesis of NA

The pathogenesis of NA remains unclear, namely the mechanisms underlying the generation of acute abdominal pain, and the development of fibrosis and hyperplasia of the nerve fibers. An important open question in this field is: How do neural hyperplasia and fibrosis develop in NA? It is the view of these authors that the alterations associated with NA are due to the increased density of the neuroendocrine ganglia and neuronal hypertrophy. This is associated with the increased contents of several mediators, such as serotonin, SP, growth-associated-protein-43 (GAP-43), and vasoactive intestinal peptide (VIP)-positive nerve fibers. There is also a concomitant increase in the perineural inflammatory cell infiltrates [7,13,28,29].

Nemeth et al. examined the nitrergic innervations in the unremarkable appendectomy specimens, those with histologically proven acute appendicitis, and histologically unremarkable appendectomy specimens from patients with a clinical presentation of appendicitis. The investigators used nicotinamide adenine dinucleotide phosphate diaphorase histochemistry and neuronal nitric oxide synthase immunohistochemistry. They measured the density of the myenteric plexus. As compared to unremarkable appendectomy specimens, significant neuronal hypertrophy, myenteric plexuses with thick nerve bundles, and abundant ganglion cells were found both in the appendectomy specimens with acute appendicitis and the histologically unremarkable appendectomy specimens from patients with a clinical presentation of appendicitis [28]. A summary of the mediators and cells potentially involved in the pathogenesis of NA is shown in Table 1. The late stage of NA is characterized by fibrosis. Although the underlying mechanisms are not clear, it is possible that this fibroproliferative process is driven by the increased expression of pro-fibrogenic factors, such as transforming growth factor1 (TGF-1) and insulin-like growth factor 1 (IGF-1) [29].

### 1.4. The Roles of NPs and Neuroplasticity in NA

Several studies support the roles of NPs (serotonin SP, GAP-43, VIP) in the development of NA [29]. A summary of these mediators is shown in Table 1.

#### 1.4.1. VIP Neuropeptide

VIP is an NP initially isolated from the intestine. It is stored by nerve fibers innervating the gastrointestinal tract. It is expressed by the appendicular tissues of NA [13,30,31]. The VIP/pituitary adenylate cyclase-activating peptide system consists of two peptides and three receptors. The latter belong to the second family of the G protein-coupled receptors. These peptides and receptors are widely distributed in the endocrine, immune, and nervous systems. They have strong anti-inflammatory and immunomodulatory properties. The anti-inflammatory effects of VIP are mediated through scavenging the free oxygen radicals, blocking the effects of the cytokines, and inhibiting the functions of the inflammatory cells. VIP also suppresses the chemokine receptors and inhibits leukocyte migration [44,45].

#### 1.4.2. SP Neuropeptide

SP, a member of the tachykinin family, was initially discovered in 1931 in equine brain and gut extracts [33,46,47]. It is expressed by a variety of cells, including neurons, epithelial and endothelial cells, T cells, macrophages, and some stem and progenitor cells. SP plays important roles in the nociceptive processes [48] and is expressed by the appendicular tissues in NA [13,30,32]. In the intestine, SP binding to its receptor neurokinin-1 alters several intestinal functions, such as colonic inflammation and fibrosis. It can stimulate the migration of colonic fibroblasts and increase their production of collagen [33], thus playing an important role in the development of fibrosis in the intestine [33,49]. 

#### 1.4.3. Serotonin and Enterochromaffin Cells

The extra-epithelial enterochromaffin cells (EC cells) are subtypes of the endocrine cells that populate the lamina propria of the appendix [36,37]. These cells are closely associated with the non-myelinated nerve fibers of the mucous plexus and are abundant in NA [50,51,52]. Serotonin, a neurotransmitter, is stored and released by EC cells located amid the enterocytes. The excess serotonin is collected by platelets and mast cells in the veins draining the gut [53,54]. In NA, the number of serotonin-positive cells in the crypts is greater than in unremarkable appendix controls. Ultrastructurally, serotonin positive cells are entrapped amid Schwann cells, cells containing NSGs [3], and S100 positive spindle cells. Kalra et al. examined the plasma serotonin levels in patients with morphologically proven acute appendicitis, patients with the acute abdomen of other etiologies, and healthy individuals. They found statistically significantly high plasma serotonin levels in patients with acute appendicitis [34].

#### 1.4.4. GAP-43 Neuropeptide

GAP-43 is a membrane-associated phosphoprotein that plays several roles in axonal growth, synaptic remodeling, and secretion of catecholamines and NPs [41,42,55]. It is expressed in the central and peripheral nervous systems. It is a marker of gastrointestinal neuronal plasticity [41,56], which is a common event that occurs during inflammatory conditions, such as appendicitis. Structurally, gastrointestinal neuroplasticity entails local tissue hyperinnervation and an enhanced expression of NPs, with subsequent activation of the neural glia. Gastrointestinal neuroplasticity is associated with alterations of the neuronal electric activities and a reduced sensory threshold. This leads to an increased sensitivity to pain, due to the excessive release of several neurotrophic factors [29]. The contribution of GAP-43 to the inflammatory process of the gut is supported by its overexpression in the nerves of the chronically inflamed pancreas [57] and in the inflamed intestinal tissue following parasitic infection [58,59,60]. The immunohistochemical examination of NPs-producing cells or NPs is not necessary for the correct diagnosis of NA.

Although previous studies have reported some case series of NA, our knowledge about the clinicopathologic features and pathogenesis of this entity is still limited. This study was performed to address these issues. 

## 2. Materials and Methods

### 2.1. Methods

This study included a PubMed literature search and meta-analysis of the studies describing the clinicopathological features of NA. It did not include any interactions or interventions with human subjects or any access to any identifiable private information. Therefore, the study did not require an institutional review board review. This study was performed in accordance with the principles of the Declaration of Helsinki [61].

### 2.2. Search Strategy and Selection Criteria

**Figure 1:**
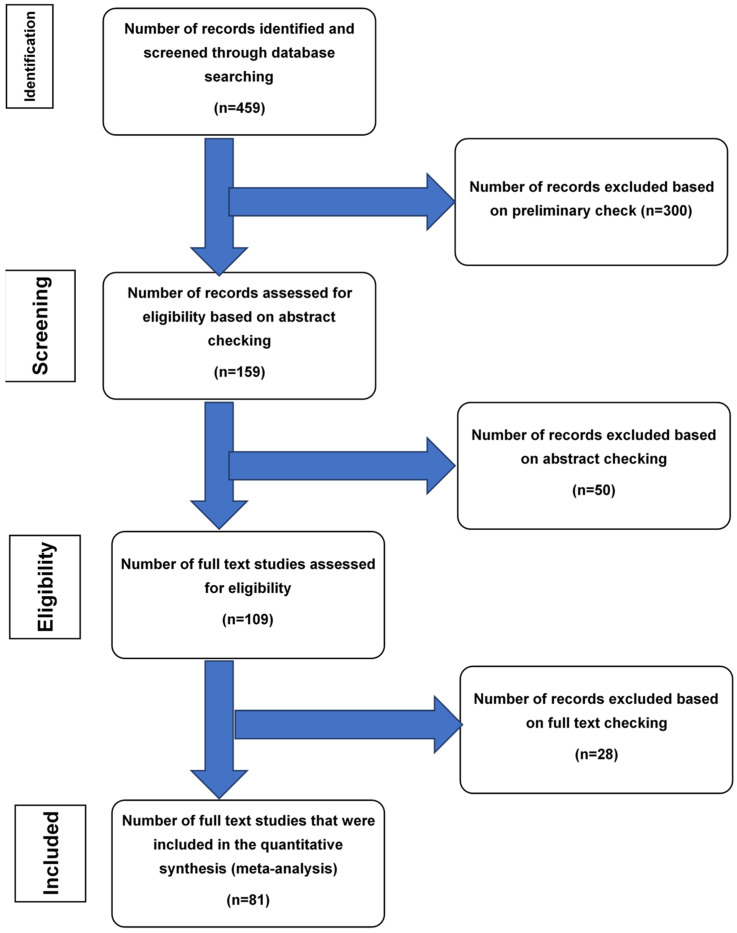
Flow chart of literature search and study selection for cases of neurogenic appendicitis.

We adhered to PRISMA guidelines (preferred reporting items for systematic reviews and meta-analysis) [62,63]. We reviewed the pertinent literature for the subject of NA by evaluating the original studies published in peer-reviewed journals. We searched the PubMed electronic database using several key terms, including “appendix” and several terms as follows: “appendix and neurogenic appendicitis,” “appendix and obliterative appendicitis,” “appendix and appendicopathy,” “appendix and neuroma,” “appendix and fibrous obliteration of the appendix,” and “appendix and chronic appendicitis” to identify the eligible studies. The eligible search results were initially considered based on their titles and abstracts. Their full texts were then comprehensively reviewed to confirm their eligibility, and if eligible, they were subsequently included in this study (Figure 1). 

### 2.3. Data Extraction

The clinicopathological features were extracted from each study. They included patient age, gender, symptoms, histology, special stains, ultrastructure, and various laboratory and radiological investigations. 

### 2.4. Methodological Assessment

The authors independently read and analyzed each study. Studies that met all of the following criteria were included in the literature analysis: (i) human studies, and (ii) full-length articles published in the English language, with the above-mentioned search keywords being included in the title or abstract as a final diagnosis. Full-length articles that did not meet all the inclusion criteria were excluded from the analysis. Moreover, duplicate publications, conference abstracts, editorial letters, comments, expert opinion papers, review articles, guidelines, consensuses, or protocol studies were excluded.

### 2.5. Statistical Analysis

Statistical computations were all performed using IBM-SPSS 21.0 (IBM-SPSS Inc., Chicago, IL, USA). 

## 3. Results

Flow trial and the PubMed literature analysis: A summary of the flow chart of the literature search and study selection is depicted in Figure 1. This search yielded 459 results (articles), of which 300 were excluded from the study for various reasons. These included language and text limitations and the lack of supportive pathological data. Finally, the remaining 159 articles about NA underwent an abstract review. These studies covered a period of 99 years (1921 to 2020). This relatively long period allowed for the proper evaluation of a significant number of cases in the literature. A total of 28 and 50 studies were excluded after checking the abstracts and full-length articles, respectively. A total of 81 cases, including 9 case series, all with the final diagnosis of NA, were included in the current analysis. Some of these previous case series are presented in Table 2.

### 3.1. Clinical Features of the Cases of NA

Analysis of the previous PubMed published cases of NA revealed several observations. NA represented 17.6% of all cases of surgically treated appendicitis in the articles that were analyzed. NA was uncommon in the first decade of life (less than 1% of cases), and most common in the second decade of life. The mean age of the patients found in the meta-analysis was 26.7 ± 1.7 years. The age distribution was as follows: 10% (<15 years), 20% (16–25 years), 70% (>26 years). NA was more common in females, with a male to female ratio of 2:3. It seems that there is a second wave of NA that develops after the age of 45 years. Currently, appendicitis in elderly people represents an increasing problem in acute abdominal surgery because of the growing occurrence [67,68].There are no available reports about the association between NA and the risk of developing acute appendicitis at an advanced age. It is possible that the development of acute appendicitis with a background of NA is related to alterations of the NPs [13,25,32].

Acute abdominal pain was the most common presenting symptom (100% of cases), followed by nausea and vomiting in 70% of cases. The patients presented with manifestations of acute appendicitis (pain in the right iliac fossa lasting from a few to several hours, with temperatures ranging from 36 °C to 39 °C. The leucocyte counts ranged from 5.0000 to 16.0000 per microliter of blood. No sufficient data were available about the proportion of patients presenting with recurrent bouts or leukocytosis. The radiological and ultrasonographic examinations were performed on some, but not all of the patients. There were no significant ultrasonographic radiological findings. The diagnosis of acute appendicitis was established clinically; therefore, appendectomies were performed. 

Our analysis revealed that there were no important differential clinical diagnostic trends between NA and acute appendicitis with typical or specific symptoms. Moreover, no specific laboratory or radiological features were found that can separate NA from acute appendicitis. The patients with NA are oftentimes treated with an emergency appendectomy, similar to cases of acute appendicitis. In NA, there are no postoperative complications following appendectomy. Alternatively, postoperative complications may occur in some cases of acute appendicitis.

### 3.2. Pathological Features of the Cases of NA

Intraoperatively, the appendices were grossly unremarkable. Their lengths ranged from 2 cm to 6 cm. Their diameters ranged from 0.3 cm to 0.5 cm. The serosal coverings were smooth and glistening. No sites of perforations or necrotic materials were identified. The walls of the appendectomy specimens had a light tan-white appearance. Their lumens were completely patent (submucosal or intramucosal variants), or partially or completely obliterated (axial or central variant). The mucosae were mildly congested. The mesoappendices had an unremarkable appearance, with no evidence of hemorrhage or necrosis. No enlarged lymph nodes were identified. 

**Figure 2:**
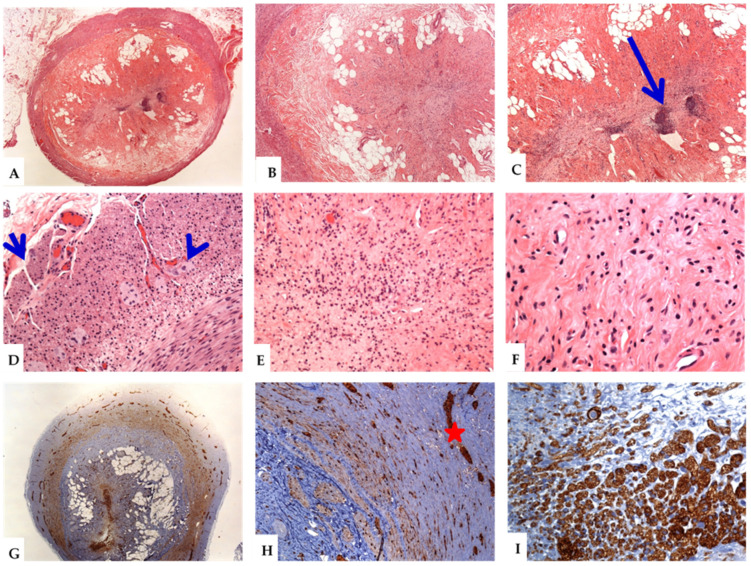
The histological features of the central variant of neurogenic appendicitis (neuroma of the appendix). Different magnifications of the appendix reveal obliteration of the lumen, effacement of the appendiceal micro-architecture, with loss of the mucosal glands and the lymphoid follicles (**A**–**F**). There is an obliteration of the lumen by residual reactive lymphoid aggregates (long arrow), and varying proportions of nerve tangles, fibrous tissue, collagen, fat, and chronic inflammatory cells (**A**–**C**). The presence of residual lymphoid cells is indicative of repeated attacks of minimal asymptomatic mucosal inflammation. Hypertrophied nerve bundles (short arrow) and ganglion cells (**D**: arrowhead) are noted. The neuronal component is composed of mitotically inactive cells, with spindly nuclei and pale cytoplasm (Schwann-like cells) obliterating the lumen and expanding the lamina propria (**E**,**F**). The hypertrophied myenteric plexuses (**G**) and nerve fibers (**H**,**I**) are stained with antibodies against S100 (**H**: red star). Synaptophysin immunostain decorates the prominent nerve plexuses, fibers, and ganglion cells (**I**). (Original magnifications, **A**: ×20, **B**: ×40, **C**: ×100, **D**: ×200, **E**: ×200, **F**: ×400, **G**: ×20, **H**: ×200, and **I**: ×400).

**Figure 3:**
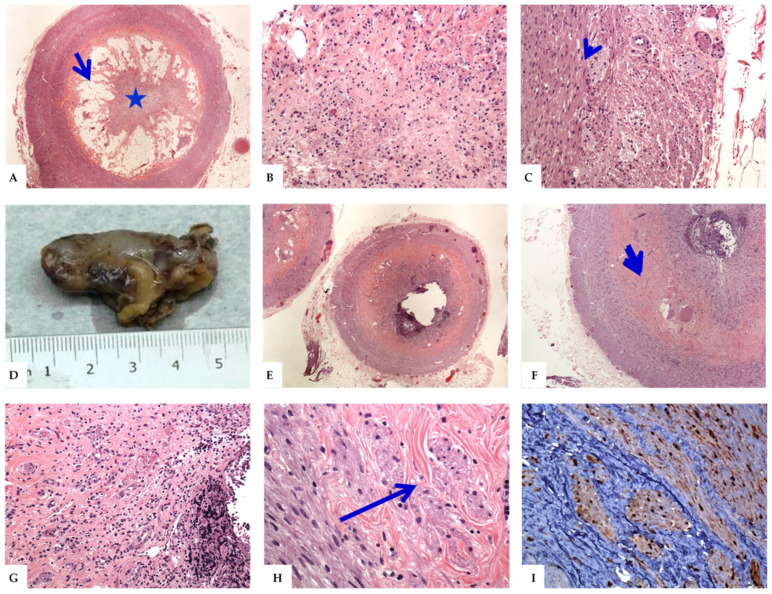
The histological features of the central and submucosal variants of neurogenic appendicitis. (**A**–**C**): Sections from the appendix showing the formation of a small central neuroma (a central variant of neurogenic appendicitis). The histological features include obliteration of the lumen by the admixture of fibrous and neural elements (**A**: star) with extensive submucosal aggregates of mature adipose tissue (**B**) and hypertrophied nerve bundles (**C**: arrowhead). (**D**)**:** Grossly, the appendix measures 3.5 cm in length ×1.0 cm in circumference with attached mesoappendix (2× 0.5 × 0.5 cm). The serosal surface is smooth and glistening. There are no perforations of the appendiceal wall or purulent materials. The mesoappendix has a yellow appearance, without areas of hemorrhage or necrosis. (**E**–**H**) On microscopy, sections reveal features of the submucosal variant of neurogenic appendicitis including extensive loss of the mucosal glands, crypts, and lymphoid follicles, associated with the expansion of the submucosa by the admixture of fibrous and neural tissues (blue short arrow) with hypertrophied nerve bundles (neurogenous hyperplasia, long blue arrow) that are reactive for S100 immunostain (**I**). The presence of lymphomononuclear cells is indicative of repeated attacks of minimal subclinical mucosal inflammation (Original magnifications, **A**: ×20, **B**: ×200, **C**: ×200, **E**: ×20, **F**: ×40, **G**: ×200, **H**: ×400, and **I**: ×200).

**Figure 4:**
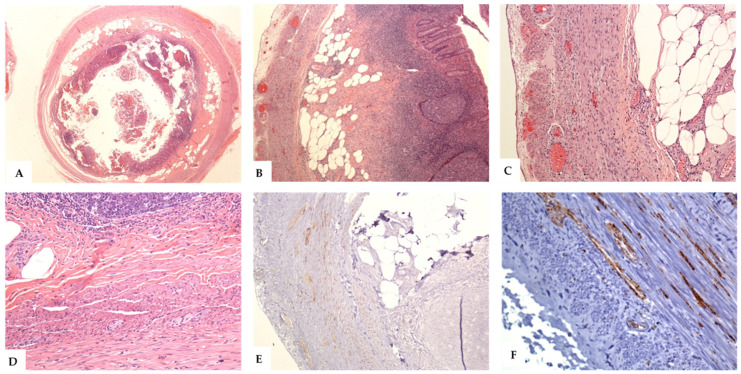
The histological features of the intramucosal variant of neurogenic appendicitis. (**A**–**D**): Sections from the appendix show intramucosal lesion with the patent lumen, destruction of the mucosal glands, and crypts expansion of the submucosa by residual lymphoid infiltrate, fibrous and neural tissues, and hypertrophied nerve bundles that extend into the mucosa. There are lobules of mature fat cells in the submucosa. The presence of lymphomononuclear cells is indicative of repeated attacks of minimal asymptomatic mucosal inflammation. No acute inflammatory cells are seen. There is no evidence of periappendicitis. (**E**,**F**): the hypertrophied never bundles and ganglion cells are consistently positive for synaptophysin. (Original magnifications, **A**: ×20, **B**: ×40, **C**: ×100, **D**: ×200, **E**: ×100, and **F**: ×400).

On histology, the salient features included the presence of regressive changes in the form of varying amounts of neurogenous and fibrous tissues, collagen fibers, mature adipocytes, and residual chronic inflammatory cells. The mucosal glands and lymphoid follicles were partially or completely lost. Immunohistologic analysis using neural markers (S100, NSE, and synaptophysin) revealed the presence of abundant neural elements and cells. There is an increase in the number and size of myenteric neuronal plexuses, prominent ganglion cells, dendritic cells, and Schwann cells. In rare cases, S100 immunostaining were performed. Staining for the neuroendocrine markers (synaptophysin or chromogranin) was not routinely performed. A summary of these findings is shown in Table 3 and Figure 2, Figure 3 and Figure 4. 

### 3.3. Histological Variants of NA

The variants of NA show some histological changes. In the axial or central variants, the changes ranged from partially preserved appendiceal lumen, with the formation of a small neuroma inside it, to complete central luminal obliteration by the fibrous and neurogenous hyperplastic tissues. The constituent cells had wavy spindly nuclei and were arranged in short bundles. There were Schwann-like cells that stained strongly for S100, synaptophysin, and NSE. The mucosal glands and crypts were usually lost. Residual lymphoid cells and variable groups of mature fat cells were seen, both in the mucosae and submucosae [3,5,9]. Hypertrophied nerve bundles and ganglion cells were seen in the appendiceal wall (Figure 2 and Figure 3). The spectrum of changes in the intramucosal variant of NA ranged from focal disruption of the mucosa, resulting in the variable loss of the mucosal glands and crypts, to separation of the mucosal crypts by neural and fibrous tissues. Some cases showed residual lymphoid aggregates, or lymphomononuclear inflammatory cell infiltrates, indicative of repeated attacks of minimal subclinical asymptomatic inflammation. These cases usually showed intact lumens. The lesional cells included proliferating spindle cells arranged in short fascicles, with elongated, wavy nuclei that extend between the crypts [4,5,14,15]. These cells were reactive to S100, NSE, and synaptophysin (Figure 3). In the submucosal variant, the lumens of the appendix were usually patent. The submucosae were variably expanded by the admixture of fibrous and neurogenous (S100 and NSE positive cells) tissues [26]. Residual lymphoid aggregates and some submucosal adipose tissues were also seen (Figure 4).

## 4. Discussion

This study was carried out to gain insights into the clinicopathologic features of NA. The novelty of the current study stems from the fact that it presented a systemic analysis of a wide scope of the reviewed literature about the clinicopathologic features of NA. It reported the evolving and regressing lesions of NA. Importantly, our study also addressed the role of neuroinflammation and the mechanisms underlying acute abdominal pain and fibrosis in NA. The main limitation of the current literature analysis was the paucity of previous studies about NA. 

### 4.1. The Early, Intermediate, and Late Phases of NA (Evolving and Regressing Lesions of NA)

Histological examination of the appendectomy specimens in NA revealed sequential phases that can be divided into early (initial, 20% of cases), active (intermediate, 40% of cases), and fibrotic (late, 40% of cases) phases. These sequential phases can only be distinguished on a histological basis. They may reflect alterations in the immune response or the neuroendocrine system. In the early phase (neurogenic appendicopathy), the appendix had an intact lumen and showed an increased number of the extraepithelial EC and Schwann cells, without significant neurogenous hyperplasia. These changes can trigger the development of neurogenic appendicopathy. Repeated minimal subclinical attacks of inflammation trigger the intermediate and late phases of NA. In the active (intermediate) phase, the appendix showed a partial obliteration of the lumen, with intramucosal neurogenous hyperplasia associated with submucosal and muscular nerve growth. The increase in the number of nerve fibers can be related to the increased release of several NPs, such as VIP and SP [13,32,69]. These changes trigger the development of the intramucosal and submucosal variants of NA. In the fibrotic phase, the fibroproliferative process resulting from the release of fibrogenic mediators can lead to the partial or complete obliteration of the appendiceal lumen. There is an admixture of fibrous and neurogenous tissues [70,71]. This final phase is characterized by the expression of S100, NSE, and synaptophysin. These changes seem to trigger the central variant of NA. During surgery, the appendix with NA appears unremarkable, with preserved lumen (early phase), or minimally congested, with dull mucosa and serosa (active phase), or with completely obliterated lumen (fibrous phase). During these phases, the appendix with NA does not have an enlarged, swollen, or purulent appearance during surgery. 

### 4.2. Neuroinflammation in NA

Neuroinflammation is a tissue response mediated by the neural cells, NPs, and yet unidentified mediators. It is involved in the development of NA [29,72,73] by inducing the hyperplasia of the neuroendocrine cells and the proliferation of the nerve fibers. These lead to an enhanced release of NPs in the wall of the appendix. Several experimental observations support this notion [7,13,26,28,29,36,73,74,75]. Vasei et al. examined the contents of serotonin in appendectomy specimens representing unremarkable appendices, those with acute appendicitis [76,77], or follicular hyperplasia, and those without histological evidence of appendicitis. The authors used antibodies against serotonin, chromogranin A, and synaptophysin. The density of EC cells was markedly reduced in acute appendicitis as compared to the other three groups (unremarkable appendectomy specimens, NA, and follicular hyperplasia). Similarly, there was a downregulation of the expression of neuroendocrine markers (chromogranin and synaptophysin) in acute appendicitis as compared to the other three groups [35]. Although the exact underlying mechanisms are unknown, this downregulation may be caused by the absence of ganglion cells and neuronal hyperplasia [35].

### 4.3. Acute Abdominal Pain in NA

Although the underlying mechanisms of pain in NA are poorly understood, the increased levels of the NPs, together with the perineural infiltration by the immune cells, seem to be involved in this process. Di Sebastiano et al. used digitized morphometry to examine GAP-43 protein expression in 29 tissue samples of chronic pancreatitis. The researchers found an upregulation of neuronal GAP-43 protein expression in pancreatic nerve fibers and intrinsic neurons. The expression values correlated with pain scores in each patient. The pancreatic nerves were infiltrated by immune cells. The authors concluded that both neuronal plasticity and infiltration of the pancreatic nerves by immune cells are involved in the development of pain in chronic pancreatitis [56]. In the appendix, Di Sebastiano examined appendectomy specimens of 15 cases of non-acute appendicitis for the alterations in peptidergic innervation for VIP and GAP-43 using immunohistochemical methods. SP was examined using digitized morphometry. The non-acute appendices were characterized by an increased expression of GAP-43 and larger amounts of SP-immunoreactive and VIP-immunoreactive nerves in the mucosal layer, as compared to appendices with acute appendicitis. There was a close spatial relationship between SP-immunoreactive and VIP-immunoreactive nerve fibers and lymphoid cells in the mantle zone of the lymph follicles [13]. The symptoms (acute abdominal pain) in NA result from neuroproliferation resulting from an increase in the neurotransmitters SP and VIP. The different expression patterns of the neuropeptides between acute and non-acute (NA)appendicitis suggest that the former is a distinct pathological entity [13]. Therefore, it is the view of some investigators that acute abdominal pain in NA is the result of neuroproliferation with overexpression of nerve fibers for PGP 9.5 in the mucosa of non-inflamed appendices, together with the abundance of neuropeptides, such as SP and VIP [13,78]. Moreover, it has been suggested that pain mimicking acute appendicitis, both in the central and intramucosal appendiceal neuroma, is due to concomitant endocrine cell hyperplasia in the adjacent uninvolved appendiceal segments [3].

### 4.4. Fibrosis in NA

In the fibrotic phase (fibrous obliteration of the appendix), the appendix exhibits lumen obliterated by varying proportions of nerve tangles and fibrous tissue [4,10,30]. Although the mechanisms underlying fibrosis are largely unknown, serotonin seems to play an important role in the development of this phase. The link between the fibroproliferative role of serotonin and the development of fibrosis was first inferred in the1960s from a carcinoid syndrome in which the neuroendocrine cells release extensive amounts of serotonin, resulting in marked tissue fibrosis in the cardiac valves [38,39,40]. In the 1980s, another link was established between retroperitoneal fibrosis and methysergide resulting from the metabolism of this compound into methylergonovine, which converts it from a 5-hydroxytryptamine 2B receptor antagonist to an agonist [79]. In liver diseases, serotonin signaling pathways can act on myofibroblasts to stimulate hepatic fibrosis. Hepatic myofibroblast serotonin (5-HT) signaling via 5-HT2B receptors enhances TGFβ1 production, which enhances fibrosis [70]. In systemic scleroderma, which is characterized by deposition of collagen in the skin, lungs, stomach, heart, and kidneys [80,81], several studies indicated that 5-HT can stimulate the proliferation of skin fibroblasts. Moreover, subcutaneous administration of 5-HT in rodents induces fibroproliferative changes reminiscent of systemic sclerosis [82].

Here, we propose that the fibroproliferative changes in the late stage of NA start by the release of 5-HT from the enterochromaffin cells, resulting in vasoconstriction with endothelial damage. The loss of anticoagulant properties of the endothelium induces platelet activation and the release of abundant amounts 5-HT, which stimulates 5-HT2B receptors enhancing TGFβ1 production, enhancing fibrosis [71]. Further experimental studies are needed to test this proposition, including the use of cell cultures(culture of appendiceal fibroblasts with 5-HT or its antagonists) and animal models. Here, we also propose that SP participates in the development of the fibroproliferative process during the late (fibrotic) stage of NA. In the intestine, SP binding to its receptor neurokinin-1 (NK-1) alters several intestinal functions, such as colonic inflammation and fibrosis. SP can stimulate the migration of colonic fibroblasts and collagen synthesis through the Akt pathway [33]. Several experimental observations support the role of SP in the development of fibrosis in the intestine [33,49]. In Crohn’s disease, there is excessive intestinal fibrosis due to excessive accumulation of fibrous tissue in inflamed areas of the small intestine and colon [33]. Koon et al. used a model of chronic trinitrobenzene sulfonic acid-induced colitis in wild-type and neurokinin-1 receptor (NK-1R)deficient mice. The authors reported collagen deposition (at the immunohistochemical level) and the upregulation of the mRNA expression of collagen and the fibrogenic factors (transforming growth factor1 and insulin-like growth factor 1) in the chronically inflamed colons of wild-type mice following the intracolonic intake of trinitrobenzene sulfonic acid. These alterations were ameliorated following administration of NK-1R antagonist CJ-12255 to the wild-type animals. The authors also found co-localization of NK-1R with fibroblasts, not only in inflamed colons of animals, but also in the large bowel mucosa of patients with Crohn’s disease [33]. The authors indicated a mechanism for SP-dependent fibrogenesis, in which SP can directly trigger the migration of human colonic fibroblasts and increase their production of collagen in combination with TGF-1 and IGF-1 through the Akt pathway [83,84]. It is conceivable that there is a similar pathogenetic process in NA.

To conclude, our study provides an in-depth review of the clinicopathological features and the pathophysiology of NA. The diagnostic criteria of NA that were applied in the original studies included the clinical features of acute appendicitis, grossly unremarkable appendix, and a lack of histological features of acute inflammation. In NA, there should be strict evidence that the acute abdominal pain in a given patient is not related to other abdominal or pelvic pathology. This evidence is based on the analysis of the preoperative clinical, radiological, and laboratory findings, along with the intraoperative gross findings [3,4,5,8,14,15,32,64,65,66,85,86,87,88,89,90]. The molecular changes in NA are open for further investigation. 

## Figures and Tables

**Table 1:** Mediators and cells involved in the generation of the neuroinflammatory response in neurogenic appendicitis.

Mediators and Cells	Sources	Mechanisms of Action	References
Vasoactive intestinal peptide	Neurons and immune cells	These G protein-coupled receptors have strong anti-inflammatory and immunomodulatory actions.	[13,30,31]
Substance P neuropeptide	Neurons, epithelial cells, endothelial cells, T cells, macrophages, some stem and progenitor cells	SP binding to its receptor neurokinin-1 alters several intestinal functions, such as inflammation and fibrosis.	[13,30,32,33].
Serotonin (5-hydroxytryptamine)	Proliferating nerve plexuses and EC cells	Serotonin plays a fibroproliferative role.	[34,35,36,37,38,39,40]
Growth-associated protein-43	Nerve fibers	It is a membrane-associated phosphoprotein involved in axonal growth, synaptic remodeling, and secretion of both catecholamines and NPs.	[13,41,42]
Pro-fibrogenic factors (transforming growth factor1 and insulin-like growth factor 1)	Endothelial cells, mast cells, and fibroblasts	Fibrosis leads to obliteration of the appendiceal lumen and effacement of the appendiceal architecture.	[29,43]

**Table 2:** Previous case series of neurogenic appendicitis.

No	Authors (Reference)	Year	Number of Cases	Reference
1	Stanley, Cherwitz et al.	1986	20	[3]
2	Olsen and Holck	1987	195	[4]
3	Guller, Oertli et al.	2001	140	[5]
4	Franke, Gerharz et al.	2002	4	[15]
5	Amber, Mathai et al.	2010	25	[64]
6	Akbulut, Tas et al.	2011	54	[65]
7	Sesia, Mayr et al.	2013	29	[32]
8	Yilmaz, Akbulut et al.	2013	134	[66]
9	Ruiz, Rios et al.	2017	8	[8]

**Table 3:** The histological, immunophenotypic, and ultrastructural features of the variants of neurogenic appendicitis.

Variants	Features	Ref.
The central variant	- It is also known as the axial variant or central neuroma. - It is the most common histological type of NA. - It usually involves the distal part of the appendix. - It is characterized histologically by effacement of the appendiceal architecture (loss of the mucosal glands, crypts, and lymphoid follicles) and the obliteration of the lumen by neurogenic tissue composed of loosely arranged aggregates of S100-positive spindle cells, blending imperceptibly into the surrounding fibrous tissue scar. - There are varying amounts of collagen, fat, and chronic inflammatory cells in the wall. - At the ultrastructural level, there is an admixture of NSGs(endocrine cells), Schwann cells, and neural processes.	[3,5,9]
The submucosal variant	- It is an uncommon variant. - It is characterized histologically by a lack of obliteration of the appendiceal lumens, in most cases. There is an expansion of the lamina propria by fibrous and neurogenous tissues.	[26]
The intramucosal variant	- It is also known as intramucosal appendiceal neuroma - It is a rare variant. - It is characterized histologically by a lack of the obliteration of the appendiceal lumen. There are mitotically inactive cells, with spindly nuclei and pale cytoplasm (Schwannoma-like cells), separating the crypts and variably expanding the lamina propria. - These cells are consistently immunoreactive for S100 proteins.	[4,5,14,15]

## Data Availability

All data are included in the manuscript. The images are original and made by the authors. They are not to be republished without permission.

## Abbreviations

NA	neurogenic appendicitis
EC cells	enterochromaffin cells
SP	Substance P
NPs	neuropeptides
NSGs	neurosecretory granules
